# Clinical characteristics of 93 cases of isolated macrodactyly of the foot in children

**DOI:** 10.1186/s13018-020-02196-2

**Published:** 2021-02-08

**Authors:** Wei Chen, Xiaofei Tian, Lu Chen, Wei Huang

**Affiliations:** 1 Department of Joint Surgery, The First Affiliated Hospital of Chongqing Medical University, No. 1 Youyi Road, Yuzhong District, Chongqing, 400016 China; 2 Department of Burn and Plastic Surgery, Children’s hospital of Chongqing Medical University, Chongqing, China; 3 Department of Ultrasound, Children’s hospital of Chongqing Medical University, No. 136 Zhongshan Er Road, Yuzhong District, 400014 Chongqing, China; 4 China International Science and Technology Cooperation base of Child development and Critical Disorders, Chongqing, China; 5 National Clinical Research Center for Child Health and Disorders, Chongqing, China; 6 Chongqing Key Laboratory of Pediatrics, Chongqing, China; 7 Ministry of Education Key Laboratory of Child Development and Disorders, Chongqing, China

**Keywords:** Macrodactyly of the foot, Clinical characteristics, Foot malformation

## Abstract

**Background:**

The purpose of this study was to describe the clinical characteristics of macrodactyly of the foot through a large cohort of cases to further understand this rare entity.

**Methods:**

Medical records, clinical photographs, plain radiographs, pathological findings, and intraoperative photographs of 95 feet of 93 patients were reviewed. Data including age; sex; laterality; ethnicity; birthplace; family history; and history of gestation, environment, whether smoking, or drinking during pregnancy were collected and analyzed.

**Results:**

Female patients (60%), left foot (56%), and static overgrowth (63%) were more prominent in the study cohort. Southern provinces (74%) and Han Chinese ethnicity (95%) predominated in terms of geographical region and demographic distribution, respectively. Multiple-toe involvement was 2.01-times more frequent than single-toe involvement. All five toes were involved with midline toes being most frequently affected overall and a medial distribution being more common than a lateral one. The forefoot was affected in 90 feet. The affected areas (toes and forefeet) were mostly located in the innervation of the affected medial plantar nerve (91%). The nerves showed enlargement in 49 feet, fatty infiltration in 25, a tortuous course in one, and were normal in 10 feet. Only six feet involved the musculature. Enlargement of phalanges and metatarsals were observed in 92 and 57 feet, respectively, and advanced bone maturation was seen in 63 feet. Twenty-two cases had syndactyly.

**Conclusions:**

Macrodactyly of the foot is a rare congenital malformation with diverse clinical manifestations and multiple elements’ involvement.

It also presents the characteristics of nerve-mediated overgrowth and “nerve territory-oriented” deformity similar to that of macrodactyly of the hand.

## Background

Macrodactyly of the foot is an uncommon congenital malformation characterized by enlargement of soft tissue and osseous elements of the foot [1] which causes many problems such as pain, calluses, ulcer, difficulty in wearing shoes, impairment in ambulatory ability and gait development, esthetic problem, and psychological issues [2]. However, the features of the clinical entity, usually reported in case reports or small case series owing to its rarity with an incidence of 1/18000 [3], are incomplete and obscure; thus, different or even contradictory results delineating the characteristics of the entities have been reported (Table 1). Moreover, macrodactyly of the feet and of the hand are considered two distinct entities [4].

**Table 1:** Published reports of macrodactyly of the feet with case numbers of 5 or more

No. of patients (feet)	Sex	Side	Type	Incidence	Neural involvement	Forefoot affected	Author	Article type
	M: F	L: R: B	P:S	Hand: foot				
7	6:1	3:4:0	4:2	Equal	None (*n* = 5)		Kalen et al. [4]	Original
7	3:4	3:4:0	2:5		Yes (*n* = 1)	5	Minguella and Cusi [5]	Original
7 (8)	2:5	4:2:1	8:0		Yes (prominent)	6	Dennyson et al. [6]	Original
	MP						Natividad and Patel [7]	Review
				Almost Equal			Natarajan et al. [2]	Review
15 (17)	8:7	5:8:2	12:5			15	Chang et al. [8]	Original
13	FP		8:5	20:13			Hardwicke et al. [3]	Original
8	5:3	4:4:0	7:1		4 (*n* = 6)		Wu et al. [9]	Original
12 (13)	8:4	7:4:1	3:10			Yes	Wang et al. [10]	Original
16 (18)	7:9	8:6:2	13:5			18	Kim et al. [11]	Original
13 (14)			10:4	3:13		7	Chen et al. [12]	Original
93 (95)	37:56	53:38:2	33:60		74 (*n* = 84)	90 (*n* = 95)	Ours	Original

*L* left, *R* right, *M* male, *F* female, *P* progressive type, *S* static type, *B* bilateral, *MP* male preponderance, *FP* female preponderance

To date, several relatively large case series with 17 feet from 15 patients [8], 18 feet from 16 patients [11], and 14 feet from 13 patients [12] with foot macrodactyly have been reported; however, these studies mainly referred to surgical treatment rather than clinical features. Lack of large cohort studies with delineating characteristics of macrodactyly have resulted in confusion regarding nomenclature (e.g., megalodactyly, macrodystrophia lipomatosa, macrodactyly fibrolipomatosis, lipomatous overgrowth or hamartoma, gigantomegaly, local gigantism, and digital gigantism) and misapplication that has prevented studies with large numbers of well-categorized patients and long-term outcome data [13, 14], in addition to poor diagnosis, treatment, and further understanding of the condition.

Macrodactyly encompasses a wide array of clinical phenotypes, and the extent of overgrowth differs greatly among patients; thus, in this condition, large cohort studies on clinical characteristics of macrodactyly of the foot are essential and urgent for further research. In this study, we analyzed 93 cases of foot macrodactyly and referred to the clinical presentations, anatomical distributions, radiological features, pathological findings, and additional diseases in order to better understand the clinical characteristics of this condition in children.

## Methods

The study protocol was approved by the ethics committee of our hospital, and written informed consent was obtained from the patients’ parents. The study protocol was in accordance with the ethical guidelines of the 1975 Declaration of Helsinki.

A total of 107 patients with macrodactyly of the foot underwent surgical treatment at our hospital between March 2011 and May 2020. Patients with diagnoses of other known overgrowth syndromes or otherwise uncharacterized syndromic presentations of lower extremity enlargement were excluded, such as Klippel–Trenaunay syndrome, Proteus syndrome, CLOVES syndrome, Ollier’s disease, Maffucci syndrome, Milroy’s disease, neurofibromatosis, and vascular anomalies [2]. Therefore, 93 patients with isolated macrodactyly of the foot were included in this study. Medical records, clinical photographs, plain radiographs, pathological findings, and intraoperative photographs were reviewed and retrospectively analyzed. Data including age; sex; laterality; ethnicity; birthplace; clinical history; as well as history of gestation, environment, and maternal smoking or drinking during pregnancy were collected. The chi-square test was used to compare proportions.

## Results

Fifty-six girls and 37 boys, with an average age of 43 months at the time of surgery (range 5–197 months), were identified (ratio 1.51:1).

In all, 95 feet were studied. Macrodactyly involved the right foot, left foot, and both feet in 38, 53, and 2 patients, respectively. There were 60 static feet and 33 progressive feet (ratio 1.82:1).

**Fig. 1:**
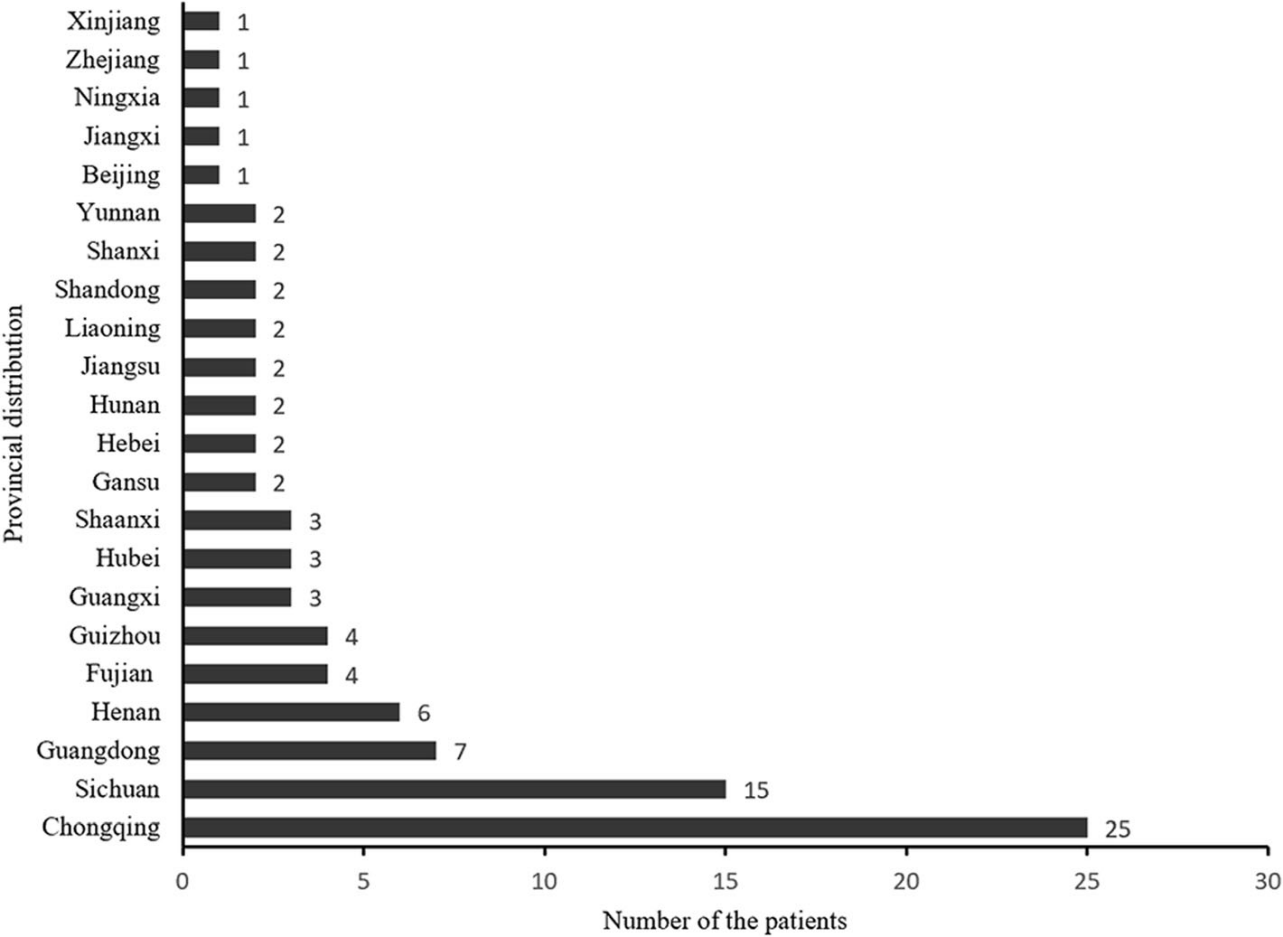
Provincial distribution of patients with macrodactyly of the foot

**Fig. 2:**
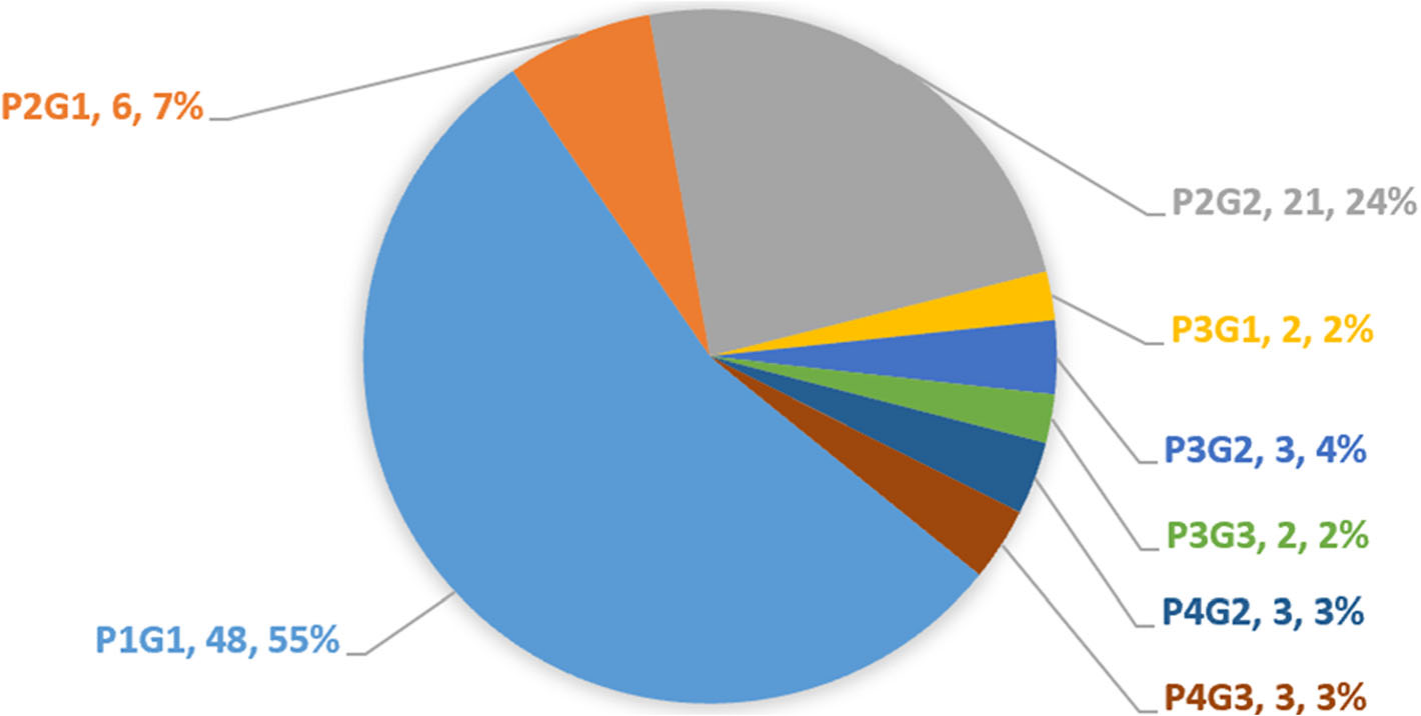
The distribution of history of gestation and delivery. P, number of pregnancies in lifetime; G, number of give births

The birth places of the 91 patients (data for two patients were unavailable) were identified across 22 provinces of China, with 12 southern provinces (69 patients) and 10 northern provinces (22 patients) (Fig. 1). In terms of ethnicity, 88 patients were Han Chinese, two were Zhuang, and one each belonged to Miao, Bai, and Yi ethnicities. There was no family history of macrodactyly or any form of overgrowth deformity. With the exception for seven mothers with upper respiratory infection, two mothers with hypertensive disorders, one mother with mastitis, and one mother with gynecologic inflammation, no history of other disease, harmful environment, smoking, or drinking during pregnancy were reported. First pregnancy and live birth accounted for 55% (*n* = 48), and multiple pregnancy and live birth accounted for 45% (*n* = 40) (Fig. 2).

**Fig. 3:**
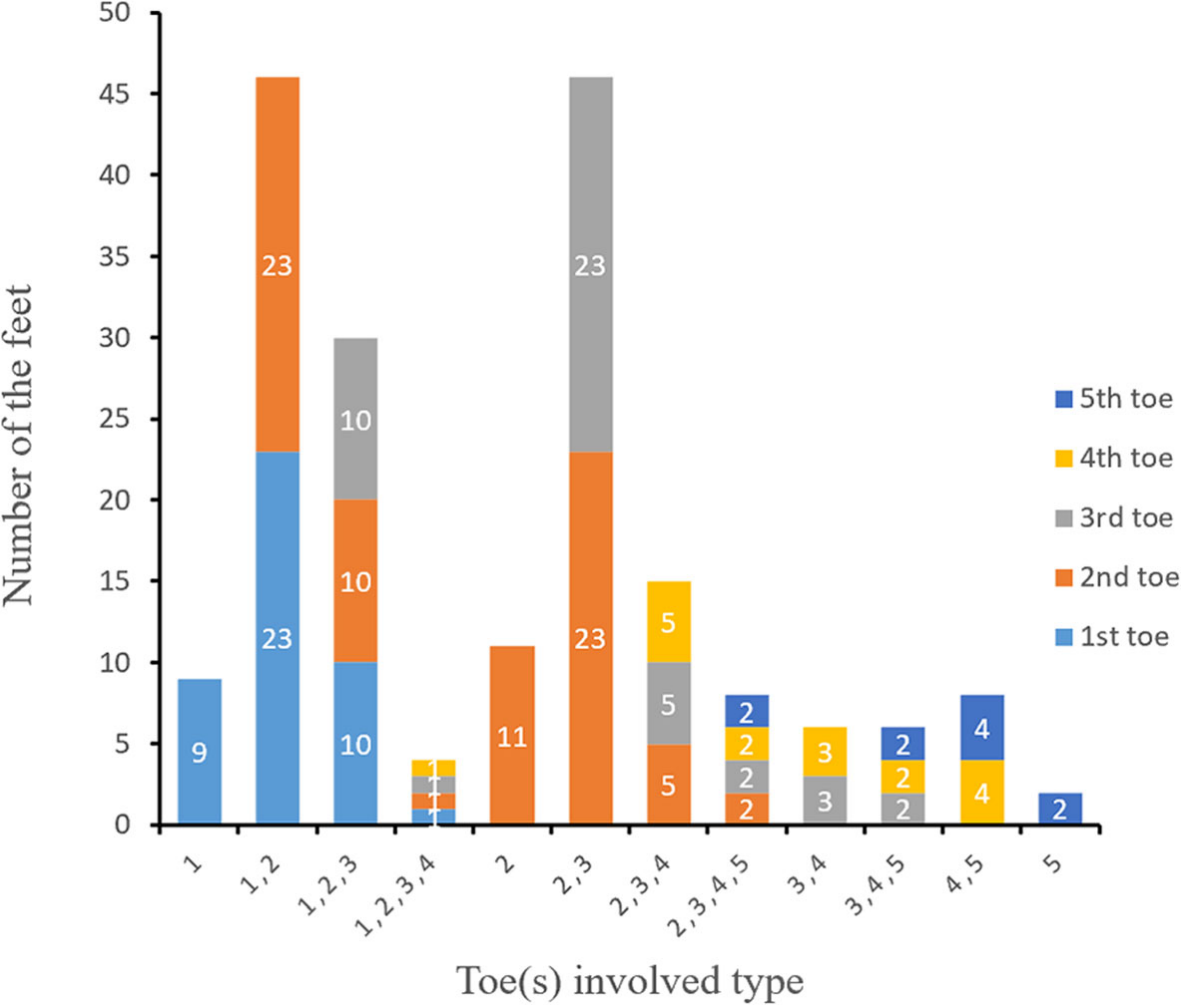
Clinical characteristics of macrodactyly of the foot. Multiple affected toes were more frequent than a single affected toe. Midline toes were mainly affected, and a medial distribution was more common than a lateral distribution

**Fig. 4:**
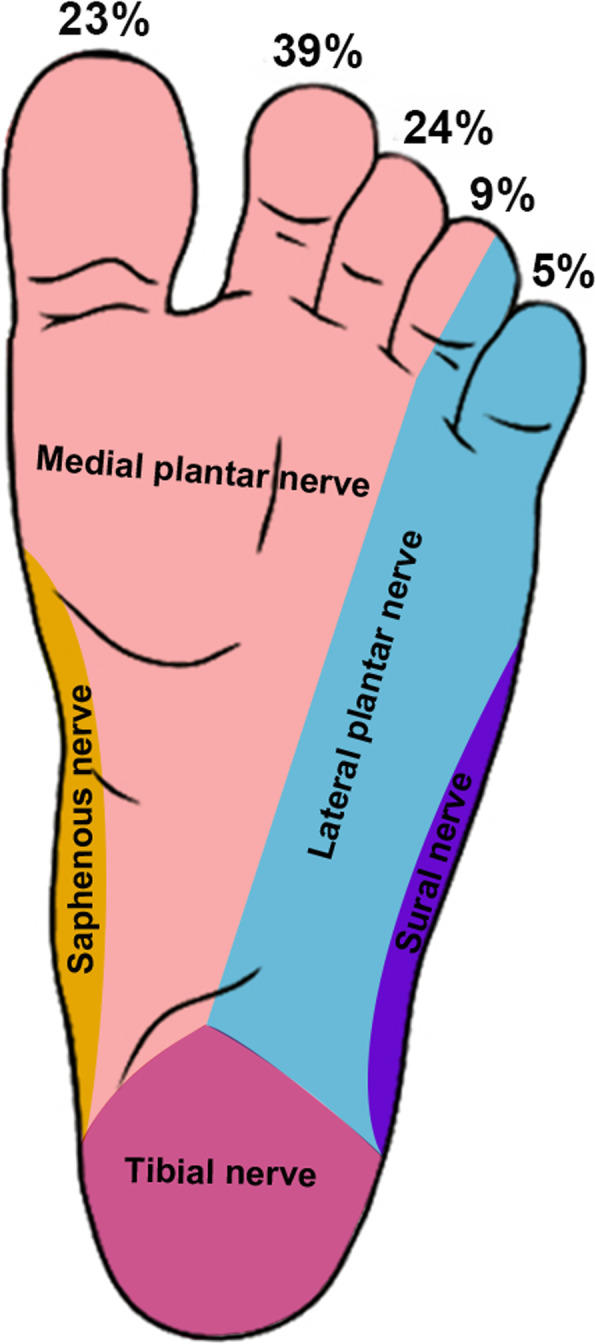
Clinical distribution of the affected toes. The second toe was most frequently involved, followed by the third, first, fourth, and fifth toe. The affected toes and forefoot were mostly located in the innervation of the medial plantar nerve

Multiple toes were more commonly affected (*n* = 73; 77%) than an isolated toe (*n* = 22; 23%), with an average of 2.01 affected toes per case. If multiple toes were involved, they were usually adjacent (Fig. 3). The incidence of affected toes varied, with the midline toes being most frequently affected overall; further, a medial distribution was more common than a lateral one. The first toe was involved in 43 feet, the second toe in 75 feet, the third toe in 46 feet, the fourth toe in 17 feet, and the fifth toe in 10 feet (Fig. 4). The forefoot along the involved toes was affected to varying degrees in 90 feet (95%). The affected toes and forefoot localized to the innervation of the medial plantar nerve were more prominent than that of the lateral plantar nerve (Fig. 4).

**Fig. 5:**
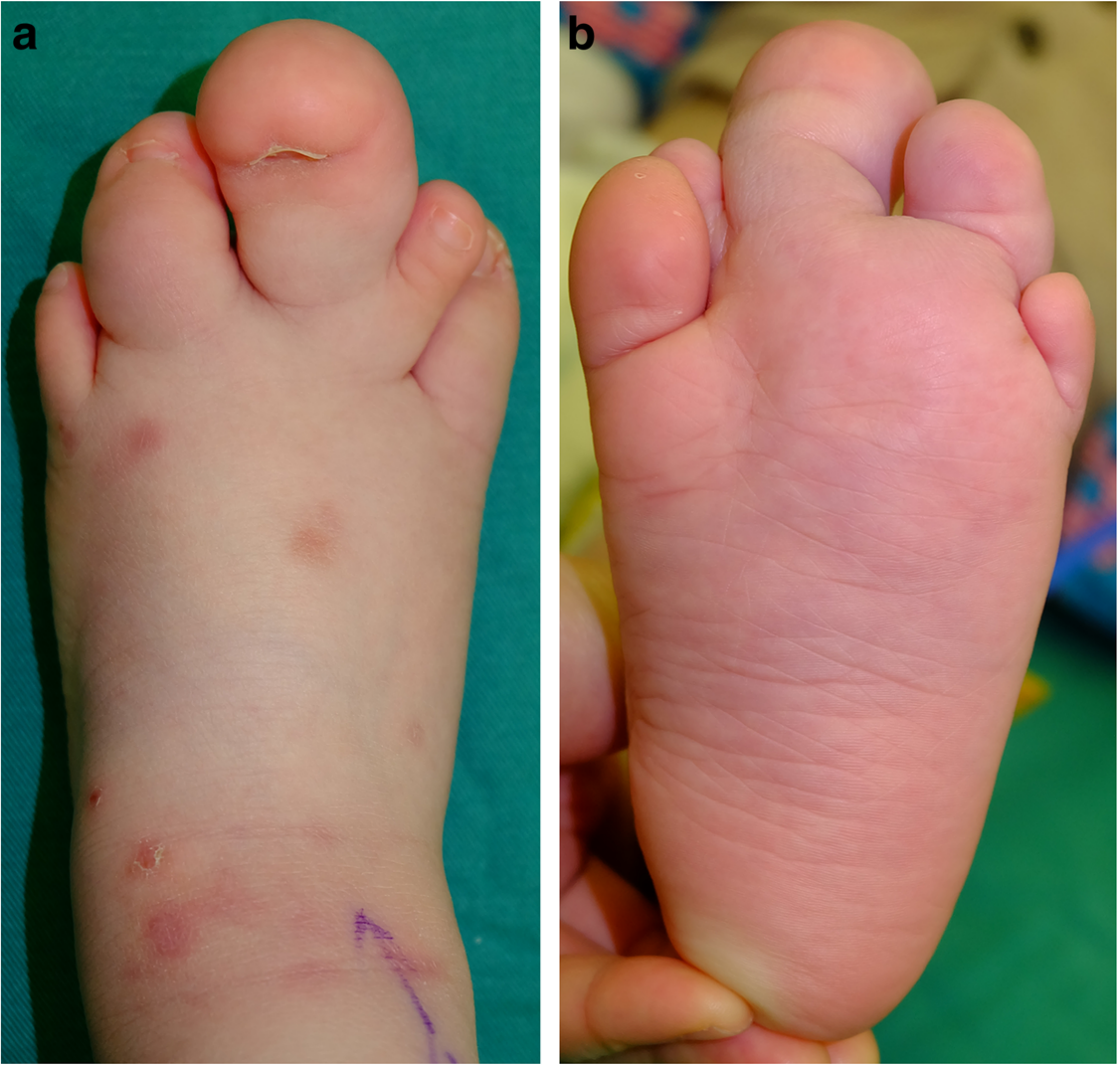
Representative picture of macrodactyly of the foot with second, third, and fourth toes involved. Asymmetrical hypertrophy on the plantar aspect of the third toe caused dorsal curling, and the dorsum of the fourth toe caused flexion contracture. Incomplete soft tissue fused between the second and the third toe (**a**). Forefoot along the involved toes was involved (**b**)

The onset time of macrodactyly varied: 82 cases (90%) at birth, one case (1%) at 1 month old, two cases (2%) at 3 months old, two cases (2%) at 6 months old, one case (1%) at 7 months old, two cases (2%) at 12 months old, and one case (1%) at 60 months old. Although, the deformity comprised an enlarged but otherwise normal-looking toe, the entire toe was not uniformly involved. The asymmetrical hypertrophy on the plantar aspect, the sides, and the dorsum in varying degree caused dorsal curling (*n* = 108; 56%), incline (*n* = 59; 31%), and flexion contracture (*n* = 25; 13%) of the affected toes (Fig. 5a).

Syndactyly is the most commonly additional malformation (*n* = 22; 23%) with the affected toes, especially syndactyly between the second and third toes (Fig. 5), followed by polydactyly of the affected toe (*n* = 4; 4%). Other additional ipsilateral deformities included polydactyly of the thumb (*n* = 1), lipoma of the knee (*n* = 1), pediatric indirect inguinal hernia (*n* = 1), and microtia syndrome (*n* = 1). Other systemic diseases included pre-excitation syndrome (*n* = 1), atrial septal defect (*n* = 1), ventricular septal defect (*n* = 1), hypospadias (*n* = 1), scoliosis (*n* = 1), and obese fatty liver (*n* = 1). No pediatric tumor was found.

**Fig. 6:**
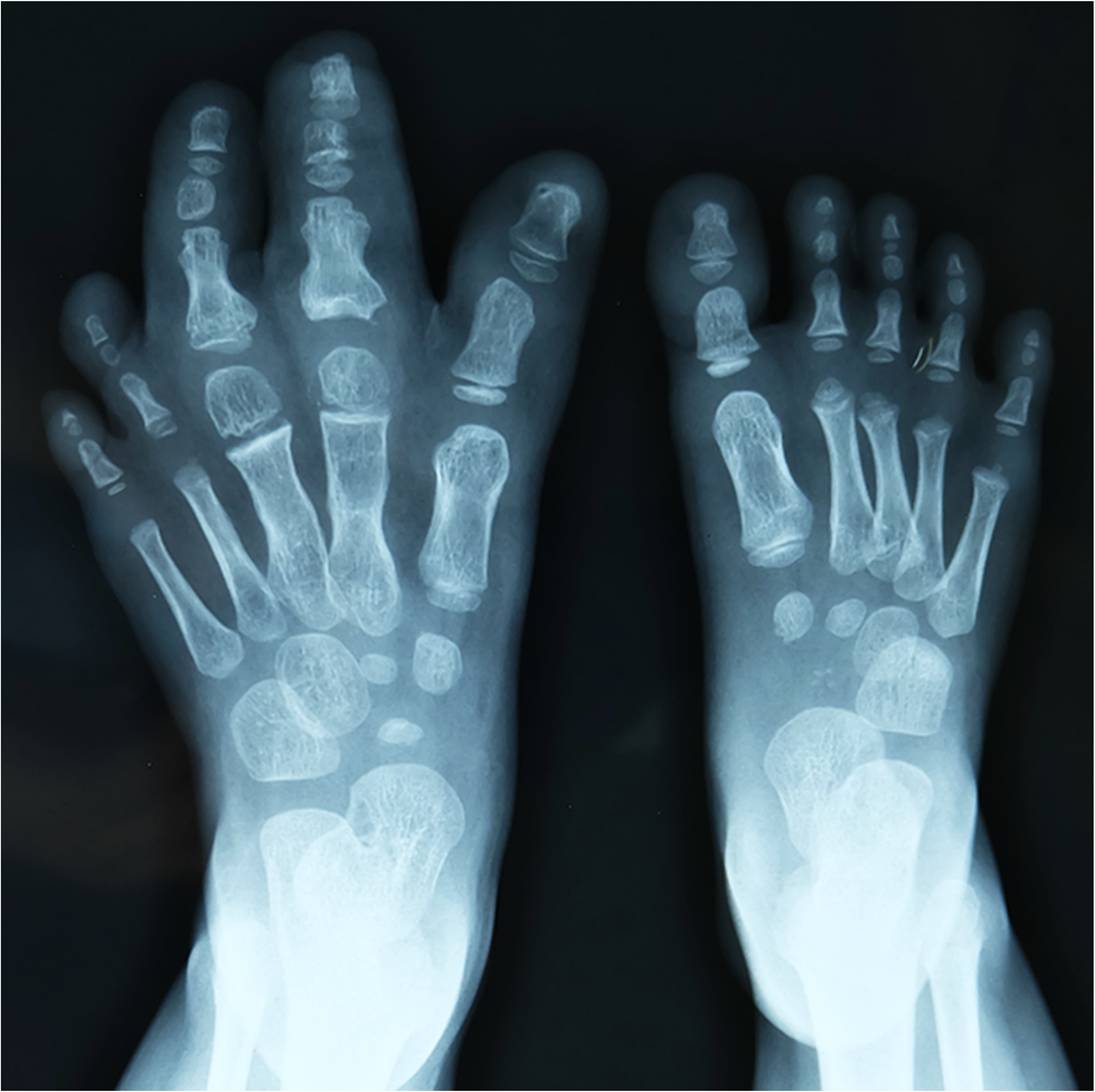
Plain radiograph showing hypertrophy of phalanges and metatarsal bones in the first, second, and third ray as well as advanced bone maturation in the second and third metatarsal capital epiphyses. The phalangeal basal epiphyses were well formed and larger than the normal foot

**Fig. 7:**
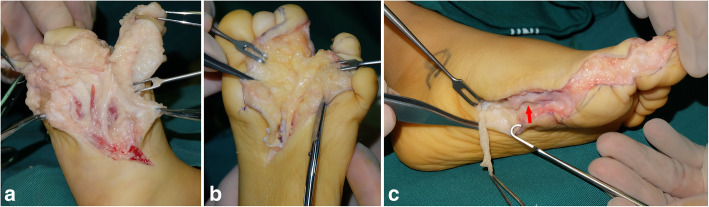
The clinical findings of the affected nerve showing enlargement (**a**), fatty infiltration in the epineurium (**b**), and tortuous course (**c**)

**Fig. 8:**
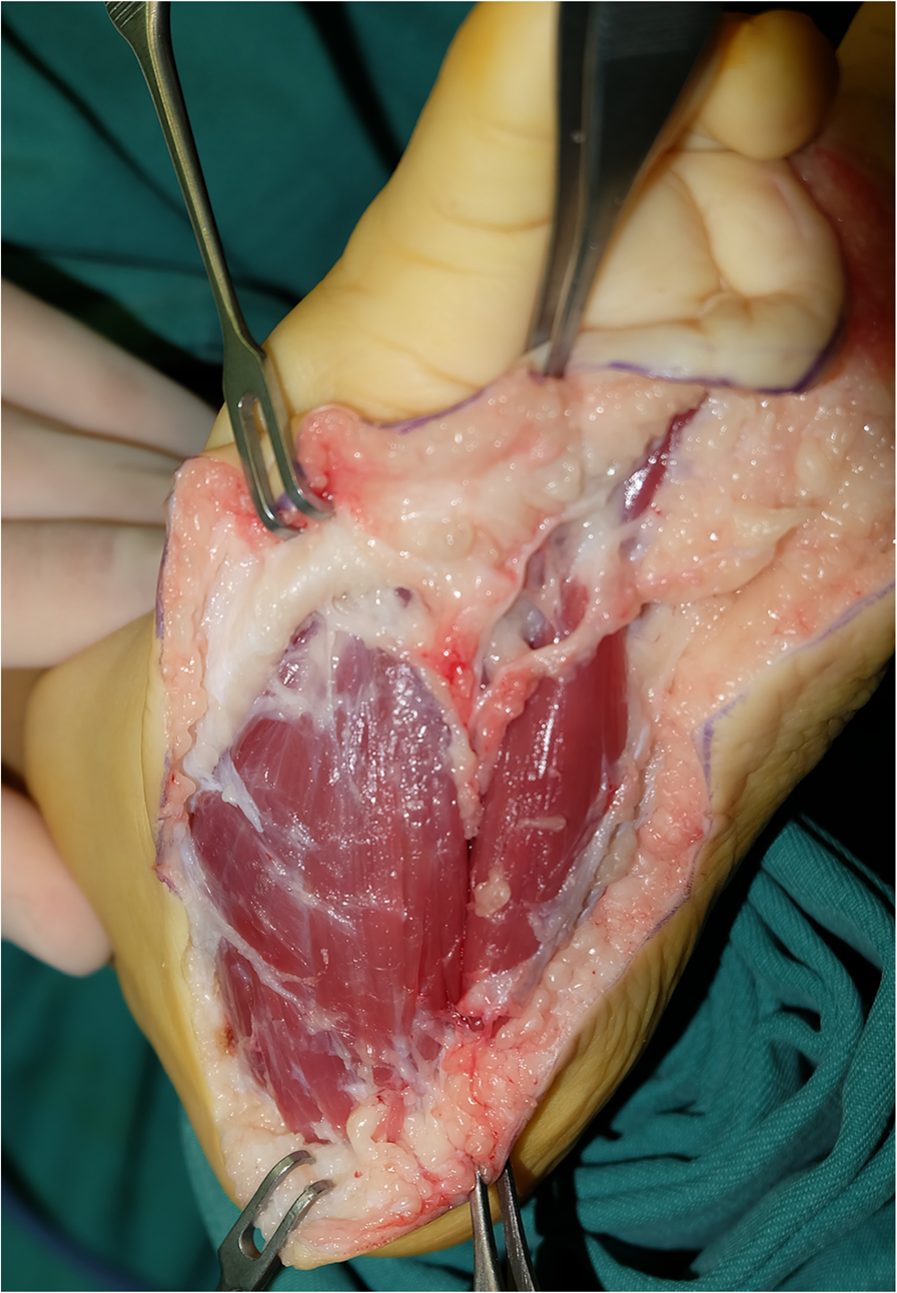
Intraoperative photograph showing hypertrophic flexor digitorum brevis

Multiple elements of the foot were affected, with fat hypertrophy being the most striking feature and extended from the toe to the forefoot causing forefoot enlargement. Bony architecture was normal in affected feet, the length and width of the phalanges in the affected toes were increased (*n* = 92; 97%), and metatarsals were enlarged in 57 feet (60%). Advanced bone maturation was found in 63 feet in terms of the metatarsal capital epiphyses, and phalangeal basal epiphyses were well formed and larger than normal counterparts (Fig. 6). The movement of interphalangeal joint and metatarsophalangeal joint decreased to varying degrees, even stiffness, owing to hypertrophy. The nerve condition was described in the surgical record of 84 feet: the nerves showed enlargement in 49 feet, fatty infiltration in 25, and a tortuous course in one foot (Fig. 7). Only six feet showed involvement of plantar muscles of the foot (Fig. 8) (Table 2).

**Table 2:** Distribution of involved structures and elements in 95 feet

Toe (s) involved type (feet)	Forefoot	Soft tissue only	Bone		Nerve				Muscle	Macrosyndactyly (feet)
			Phalanx	Metatarsal	Enlargement	Fatty infiltration	Normal	Tortuous course		
1 (*n* = 9)	9		9	5	4	3		1		
1, 2 (*n* = 23)	23		23	13	12	7	2		2	1, 2 (*n* = 1)
1, 2, 3 (*n* = 10)	10		10	10	4	5	1			2, 3 (*n* = 1)
1, 2, 3, 4 (*n* = 1)	1		1	1			1			
2 (*n* = 11)	11		11	1	5	4				
2, 3 (*n* = 23)	20	1	22	19	12	4	4		2	2, 3 (*n* = 17)
2, 3, 4 (*n* = 5)	5		5	4	4					2, 3, 4 (*n* = 2)
2, 3, 4, 5 (*n* = 2)	2		2	1	1	1				
3, 4 (*n* = 3)	2	1	2	0	1	1	1			3, 4 (*n* = 1)
3, 4, 5 (*n* = 2)	2		2	1	2				1	
4, 5 (*n* = 4)	3	1	3	1	3		1		1	
5 (*n* = 2)	2		2	1	1					
Total	90	3	92	57	49	25	10		6	22

A total of 77 pathological reports were available, and results included aggressive overgrowth of fibrofatty tissue, mature adipose tissue, enlarged peripheral nerves wrapped around by fibrofatty tissue, lipid droplet invasion, and fibrosis only in thickened epineurium of the affected plantar nerve.

## Discussion

This report described the characteristics of macrodactyly of the foot in a large cohort of 93 patients, and presented some new features on this relatively rare entity. The study indicated a preponderance for female sex, left-foot, and static type (Table 1). Pedal macrodactyly mostly involved the progressive type in other studies (Table 1); however, static type preponderance in this study might be on account of our large cohort of cases which revealed the natural growth feature of the entities. Another reason was that disproportionate progressive overgrowth of toes frequently started from the age of 2 years onward [15] or early childhood [2, 16], but patients under 2 years old in the static type group accounted for 45% (*n* = 48) cases. Therefore, it is likely that a few “real” progressive type cases may not have presented in a timely manner.

Although, none of our patients had a positive family history or any similar deformity consistent with other reports [7, 17] and the external environmental factor did not seem to affect the pathogenesis of the pedal macrodactyly, multiple pregnancy and live birth accounted for 45%, indicating that intrauterine environment might play an important role in the pathogenesis of foot macrodactyly during fetal development [1, 18].

Multiple toes were affected more often than an isolated toe, two toes type affected mostly (Table 2), and the ratio of involvement of multiple digits was higher in the foot than in the hand [4, 14]. The second toe showed the highest incidence of being affected, followed by the third, first, fourth, and fifth according to the distribution characteristics of the hand [3]. Though toe enlargement was the most immediately obvious feature, involvement of the forefoot was often overlooked (Fig. 5b), which might have been the reason for poor surgical results and additional treatment [8, 19]. The affected areas (toes and forefeet) were mostly located in the innervation of the affected medial plantar nerve (91%) (Fig. 4) indicating a close relationship between these anatomic entities. Thus, foot macrodactyly presented characteristics of being “nerve territory-oriented” in terms of the clinical and anatomic features being similar to the affected digits mainly located in the territory of the median nerve in hand macrodactyly [13, 14]. Seventy-four of 84 nerves presented pathologic changes including enlargement, fatty infiltration, tortuosity, or a combination of these [20]. The highly consistent distribution of the nerve abnormality and the tissue hypertrophy indicated macrodactyly of the foot as being a nerve-mediated overgrowth [12, 20].

Enlargement of the osseous element was an integral feature of macrodactyly [1], while increase in the length and width of the phalanges of the affected toes accounted for 97% which was slightly lower than that of phalanges of the affected fingers (100%) [14]. Enlarged metatarsal bone accounted for 60% in this study, compared to 71% (*n* = 17 feet) [8], 100% (*n* = 18 feet) [11], and 79% (*n* = 14 feet) [12] in the foot and was more prominent than enlarged metacarpal bones of the hand (11%) [14]. Macrosyndactyly was present in 22 cases (23%) in this series, which was more than that in the hand (7.8%) [14], accompanied with the second webspace being mostly involved.

Macrodactyly of the foot could be accompanied by other systemic deformities with local or general occurrence. An interesting finding was that if an asymmetrical problem was referred, the deformities often appeared on the same side as the macrodactyly. Although considered a benign overgrowth [21], the pathogenesis of isolated macrodactyly is related to somatic *PIK3CA* mutations [22, 23]. However, activating *PIK3CA* mutations was also responsible for a variety of tumors [24, 25]. Although no tumor had been found in our study and other reported cases with macrodactyly, given the same pathogenesis between macrodactyly and tumors and inadequate evidence to demonstrate its risk, it may be prudent to consider periodic ultrasound examination on patients with macrodactyly.

## Conclusions

We describe the characteristics of foot macrodactyly in a large cohort of 93 cases as well as present some rare clinical features, which bring us closer to understanding the entities. However, we acknowledge that our study has some limitations: (1) all data being from one single center may have introduced bias in the analyses and (2) sensory disturbance and temperature changes were not reported as part of complete clinical characteristics because it is difficult to get an accurate sensory response in children and all of our cases were children.

## Data Availability

The datasets used and/or analyzed during the current study are available from the corresponding author on reasonable request.

## Abbreviations

CLOVES: Congenital lipomatous overgrowth, vascular malformations, epidermal nevi, scoliosis/skeletal and spinal
PIK3CA: Phosphatidylinositol-4,5-bisphosphate 3-kinase catalytic subunit alpha
L: Left
R: Right
M: Male
F: Female
P: Progressive type
S: Static type
B: Bilateral
MP: Male preponderance
FP: Female preponderance
